# 

**Published:** 1888-03-03

**Authors:** 


### CONTENTS.

**Huxley's Way of Life** - - - - 375

**Words of Consolation.** Chapter LXVIII.—The Sufferings of Christ - - - - 376

**The Doctor's Pulpit.**—An Apology for the East Wind - 377

**The Glasgow Hospital for Sick Children** - - 378

**The Countess of Dufferin's Fund** - - - 378

**Aids to Nursing.** By M. M. Basil, M.A. M.B., Author of "The Commoner Accidents to Life and Limb" - 379

**The Norwich Collection of Calculi** - - - 380

**Scotch Physicians and Surgeons.**—By C. G. Furley.—Sir Robert Christison - - 381

**Notes and News** - - - - 382

**Everybody's Page** - - - - 384

**Annotations.**—Bricks or Daisies?—Doctors in Cotton Wadding.—Home Colonisation - - - 385

**Amusements for Convalescents.**—Palmistry Notes. By a Lady - - - 386

**Rachel Challice's Vow.** By Lina Nevill, Author of "A Future on Trust," etc. - - - 387

**The Common Accidents of Every-day Life.** By a Surgeon. Fractures and Dislocations - - 389

**The Nursing Mirror.**—Homes Abroad for Nurses.—Employment in India.—Winter Nursing Engagements - - 389

**Editor's Letter Box.** Small-pox Cases at a Public Meeting 389

**Amusements with Profit for all Ages.**—For Men and Women.—Children's Corner.—Puzzles, etc. - 390

